# Remote AI Screening for Parkinson’s Disease: A Multimodal, Cross-Setting Validation Study

**DOI:** 10.21203/rs.3.rs-6844936/v1

**Published:** 2025-06-26

**Authors:** Md Saiful Islam, Tariq Adnan, Abdelrahman Abdelkader, Zipei Liu, Evelyn Ma, Sooyong Park, Asif Azad, Pai Liu, Meghan Pawlik, Emily Hartman, Erin Shelton, Kristina B. Larson, M Saifur Rahman, Cathe Schwartz, Karen Jaffe, Jamie L. Adams, Ruth B. Schneider, Jan Freyberg, E. Ray Dorsey, Ehsan Hoque

**Affiliations:** 1 Department of Computer Science, University of Rochester, Rochester, New York, United States.; 2 Department of Computer Science and Engineering, Bangladesh University of Engineering & Technology, Dhaka, Bangladesh.; 3 Department of Neurology, University of Rochester Medical Center, Rochester, New York, United States.; 4 InMotion, Beachwood, Ohio, United States.; 5 Department of Global Health and Social Medicine, Harvard Medical School, Massachusetts, United States.; 6 Health AI, Google Research, London, United Kingdom.; 7 Health Services, Ministry of Defense, Riyadh, Saudi Arabia.

**Keywords:** AI screening, Parkinson’s disease, Parkinsonian disorders, movement disorders, remote assessment, artificial intelligence, home assessment

## Abstract

PARK is a web-based artificial intelligence (AI) tool for remote screening of Parkinson’s disease (PD) using video and audio recordings of speech, facial expression, and motor tasks performed via webcam. The study draws on data from 1,865 participants across diverse global demographics and recording environments, including supervised clinical settings and unsupervised home use. On three independent test sets (n=389; 188 with PD), PARK achieved strong predictive performance on classifying individuals with and without PD, with accuracy ranging from 80.2% to 80.6% and area under the receiver operating characteristic curve (AUROC) from 0.85 to 0.87. When evaluated on 30 randomly selected individuals, PARK’s assessments were 83.7% accurate and showed high agreement with three movement disorder specialists. The model generalized well across age, sex, and ethnic subgroups and incorporated mechanisms to withhold uncertain predictions to support safe use in unsupervised settings. Usability studies confirmed high participant satisfaction and preference for remote screening. These findings support the potential of PARK as an accessible, scalable, and clinically aligned tool to identify individuals with PD when access to traditional healthcare settings is scarce.

Parkinson’s disease (PD), one of the fastest-growing neurological disorder [1], imposes a substantial and growing economic burden, with current estimated costs in the U.S. exceeding $50 billion [2]. Yet timely detection remains out of reach for many [3, 4], as diagnosis typically relies on in-person assessments that are often costly, time-consuming, and inaccessible—particularly in underserved regions [5, 6]. Even where care is available, long wait times and limited specialist access delay diagnosis and intervention [7, 8]. As timely intervention offers a chance to preserve quality of life [9], there is an urgent need for scalable, non-invasive screening tools to reach individuals before symptoms become disabling.

Several approaches have emerged to facilitate PD assessment outside the clinic, including wireless and wearable sensors [10–13], speech-based tools [14, 15], and video analysis [16–21]. While these methods show promise, they are often constrained by cost, complexity, or limited scope—typically relying on a single task or modality. Biomarker-based diagnostics, such as the cerebrospinal fluid *α*-synuclein seed amplification assay [22], have demonstrated high accuracy but are invasive and logistically challenging for broad deployment. Many studies also draw from small, demographically narrow cohorts, limiting generalizability. Given the heterogeneity of PD symptoms [23], multimodal tools that are robust, accessible, and scalable are essential to capture the full spectrum of PD manifestations.

In prior work, we introduced UFNet [24], a lightweight multimodal model that analyzes short webcam recordings of three standardized tasks—speech, facial expression, and finger tapping—to screen for PD. In this study, we advance UFNet from a promising algorithmic framework to a clinically grounded, user-centered screening tool named Parkinson’s Analysis with Remote Kinetic-tasks (PARK) by conducting a multi-pronged evaluation across three independent test sets. First, we assess PARK on a demographically balanced cohort to evaluate generalizability and fairness. Second, we report findings from two prospective validation studies conducted in two distinct real-world contexts: a supervised community setting and an unsupervised home-use setting, each designed to evaluate model performance and user experience. These studies incorporate structured surveys to capture participants’ perceptions of usability, psychological comfort, and the perceived benefits and limitations of the tool. Finally, we benchmark PARK’s predictions against the assessments of three expert neurologists on a subset of external participants to contextualize the model’s clinical relevance. Together, these contributions provide a rigorous foundation for assessing the readiness of AI-enabled, at-home PD screening. More broadly, this work offers a model for evaluating the safety, usability, and equity of digital diagnostic tools. We present an illustrative overview of the PARK tool and the corresponding datasets used in this study in Fig. 1.

## Results

### Study Participants

Participants were recruited across eight independently conducted studies, each approved by the corresponding institutional review boards. Recruitment occurred via diverse channels, and participants recorded standardized task videos in varied environments. Two studies (Parktest and Validation Study-2) were conducted entirely remotely, with participants independently completing recordings from home. Two other studies (Parktest@InMotion and ROUTE-PD) took place at a PD wellness center with on-demand (available if needed) staff supervision. The remaining four studies (SuperPD, ROOSTER-PD, Cluster-PD, and Validation Study-1) were supervised by the study team, though some components were conducted remotely due to COVID-19 constraints. Detailed descriptions and study-specific inclusion/exclusion criteria are provided in the Methods section.

**Fig. 1:**
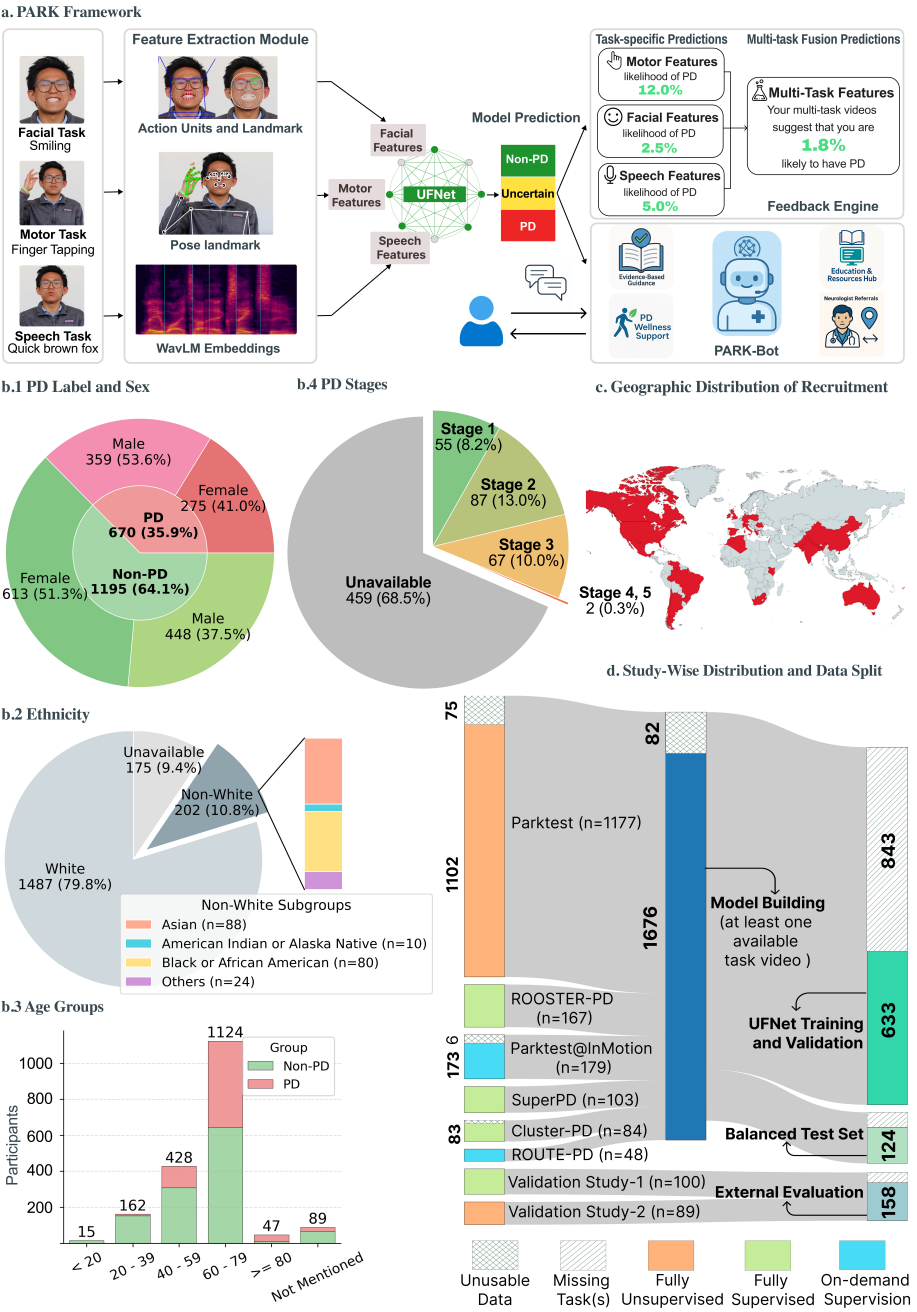
Study overview. (a) Overview of the PARK screening tool. Users complete three standardized tasks—facial expression, finger-tapping, and speech—via webcam. Landmark trajectories and speech features are extracted using MediaPipe [25], OpenFace [26], and WavLM [27], and summarized into clinically relevant features. Task-specific neural networks estimate PD risk and uncertainty, which are integrated by the UFNet fusion model to generate an overall prediction. The system withholds uncertain outputs and provides task-level assessments, educational resources, and a large language model-powered chatbot for user feedback and support. (b) Demographic characteristics of study participants, including: (b.1) joint distribution of sex and PD diagnosis; (b.2) ethnicity distribution; (b.3) age distribution; and (b.4) distribution of Hoehn and Yahr Parkinson’s disease stages. (c) Geographic distribution of study participants. Countries highlighted in red represent locations from which participants contributed data, illustrating the global reach of the study cohort. (d) Dataset composition and study splits. Participants from six independent studies were used to construct the training, development, and balanced test sets, with strict separation of individuals across splits. Two additional studies, comprising entirely new participants, were reserved for external evaluation of the PD screening model. The color coding of the bars on the left indicates the level of supervision involved in data collection across the studies. Double cross-out grids represent cohorts that did not contribute any usable video data. Single cross-out grids indicate participants who provided at least one usable task video but did not complete all three tasks within a single session. Data from these participants were still used to train task-specific models. Only participants who completed all three task videos in at least one session were used for UFNet training and evaluation.

Of the 1,947 individuals enrolled, 82 did not contribute usable data (corrupted or ill-formatted video) and were excluded, resulting in a final cohort of 1,865 participants. Among them, 670 (35.9%) had PD, including 380 with clinically confirmed diagnoses and 290 who self-reported to have PD. The cohort was demographically diverse (Fig. 1b), with 10.8% (*n* = 202) identifying as Non-white and 52.1% (*n* = 972) as female. The cohort included 167 (40 already diagnosed with PD) known carriers of the LRRK2 G2019S variant, which is an autosomal dominant cause of PD. Participants represented 36 countries and ranged in age from 18 to 93 years (median = 63). Note that demographic information and country of residence were self-reported and occasionally missing. Among those with a documented PD stage (Hoehn and Yahr scale) (211 out of 670 participants with PD), 142 (21.2%) had mild to moderate symptoms (stages 1–2), and 67 (10.0%) had moderate impairment (stage 3).

Data from six studies were used to construct the training, development, and internal evaluation sets for our machine learning model (UFNet), ensuring no participant overlap across splits (Fig. 1d). The internal evaluation cohort (Balanced Test Data) represented balanced demographic subgroups in terms of PD status (50.0% with PD), sex (51.0% female), and ethnicity (25.0% Non-white). Two other studies, Validation Study-1 and Validation Study-2, were reserved for external evaluation in two use-case environments: a supervised community-based setting (External Evaluation (supervised)) and an unsupervised home-based setting (External Evaluation (unsupervised)). These two studies also gathered participant feedback on usability, utility, and perceived risks and benefits of the PARK tool.

Of the 1,865 participants with usable data, 910 (300 (33.0%) with PD) completed all three standardized tasks and provided analyzable data across all task modalities. The remaining 955 either skipped one or more tasks or had recordings that failed automated quality checks (see Methods for details). Participants from SuperPD, ROOSTER-PD, Cluster-PD, and ROUTE-PD often contributed data across multiple sessions, with each session defined as the three task recordings completed together. While sessions with missing tasks were used to train the task-specific models (if participants were included in the training set), only sessions with complete data were used to train (799 sessions) and evaluate (320 sessions) the UFNet fusion model.

### Predictive Performance

**Fig. 2:**
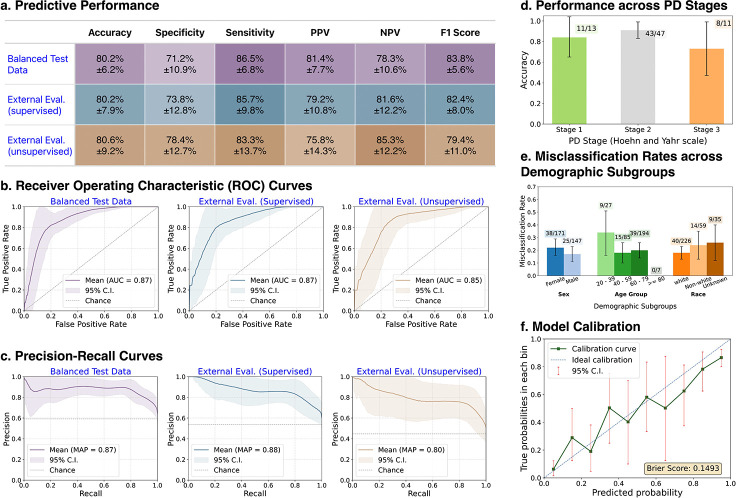
Predictive performance and subgroup analysis of the PARK tool. (a) Summary of classification metrics across internal (Balanced Test Data) and external evaluation cohorts (External Evaluation (supervised) and External Evaluation (unsupervised)), demonstrating consistently high accuracy, sensitivity, and *F*_1_ scores. (b) Receiver Operating Characteristic (ROC) curves and (c) Precision-Recall curves illustrate strong discriminative performance, with mean area under the ROC curve (AUROC) ranging from 0.85 to 0.87, and mean average precision (MAP) between 0.80 and 0.88 across the three evaluation settings. (d) Accuracy across PD stages based on the Hoehn and Yahr scale, with the highest performance observed in individuals at stage 2. (e) Misclassification rates stratified by sex, age, and race. While some variability is noted, no subgroup demonstrates disproportionately elevated error rates. (f) Calibration curve showing alignment between predicted probabilities and observed outcomes (Brier Score = 0.1493), indicating reasonable model calibration. All error bars represent 95% confidence intervals (CI).

PARK demonstrated consistent performance in distinguishing individuals with and without PD across three evaluation settings. Sensitivity remained stable, achieving 86.5 ± 6.8% (95% CI (confidence interval)) on the Balanced Test Data cohort and 85.7±9.8% on the External Evaluation (supervised) cohort. However, both sensitivity and positive predictive value (PPV) were lower in the External Evaluation (unsupervised) cohort (83.3 ± 13.7% and 75.8 ± 14.3%, respectively, see Fig. 2a for reference), and pairwise statistical comparisons revealed that most of these differences were significant. Specifically, Mann–Whitney U tests showed that PPV in External Evaluation (unsupervised) was significantly lower than in both the Balanced Test Data and External Evaluation (supervised) cohorts, and sensitivity was significantly lower compared to Balanced Test Data (*p* < 0.001 for all three comparisons). No significant difference in sensitivity was observed between External Evaluation (supervised) and External Evaluation (unsupervised) (*p* = 0.38). Notably, the model achieved its highest specificity and negative predictive value (NPV) when evaluated on data collected without supervision (External Evaluation (unsupervised) cohort). Detailed performance metrics with precision-recall and receiver operating characteristic (ROC) curves are shown in Fig. 2.

Among the 320 evaluation sessions, 71 were from individuals with documented PD stage (Hoehn and Yahr scale). PARK achieved predictive accuracies of 84.2% (65.3–100%), 91.4% (83.4–99.4%), and 73.0% (46.8–99.2%) for participants at stage 1 (mild symptoms), stage 2 (mild-to-moderate symptoms), and stage 3 (moderate symptoms), respectively. Although the model generally performed better on participants with mild and mild-to-moderate symptoms, we did not detect a statistically significant difference in predictive accuracy across stages (test statistic: *χ*^2^ = 2.94, *p* = 0.157, using Monte Carlo-simulated chi-square test).

We further assessed model performance across demographic subgroups, combining data from all three evaluation cohorts. The PARK tool showed slightly lower accuracy for under-represented groups, including female participants (77.8 ± 6.7%) and Non-white individuals (76.3±11.3%). However, no subgroup based on sex or race exhibited a significantly higher misclassification rate (*p* = 0.245 for sex and *p* = 0.293 for race; using Z-tests for proportions). Additionally, a chi-square test found no significant variation in error rates across age groups (*p* = 0.206). To account for the increased risk of Type I error from multiple tests on the same dataset, we conducted a multivariable logistic regression with false discovery rate (FDR) correction, which revealed no statistically significant demographic variables (all FDR-adjusted *p* ≥ 0.19).

Beyond binary classification, PARK tool provides a continuous score between 0 and 1, that could be interpreted as the likelihood of having PD. We evaluated the calibration of these likelihood scores using calibration curves and the Brier score [28]. PARK achieved a Brier score of 0.149, indicating moderate calibration. Likelihood estimates below 0.10 or above 0.80 were well aligned with true outcome probabilities (Fig. 2f). However, calibration was less reliable in the mid-range (between 0.3 and 0.6), where predicted scores deviated more from observed probabilities.

### Consistency with Clinician Evaluation

To benchmark PARK’s predictions against expert clinical judgment, three neurologists specializing in PD independently reviewed videos from 30 randomly selected individuals drawn from the evaluation cohorts, all with confirmed PD diagnoses (see Methods for details). Each clinician assessed whether the participant exhibited Parkinsonian symptoms based solely on the video recordings.

**Fig. 3:**
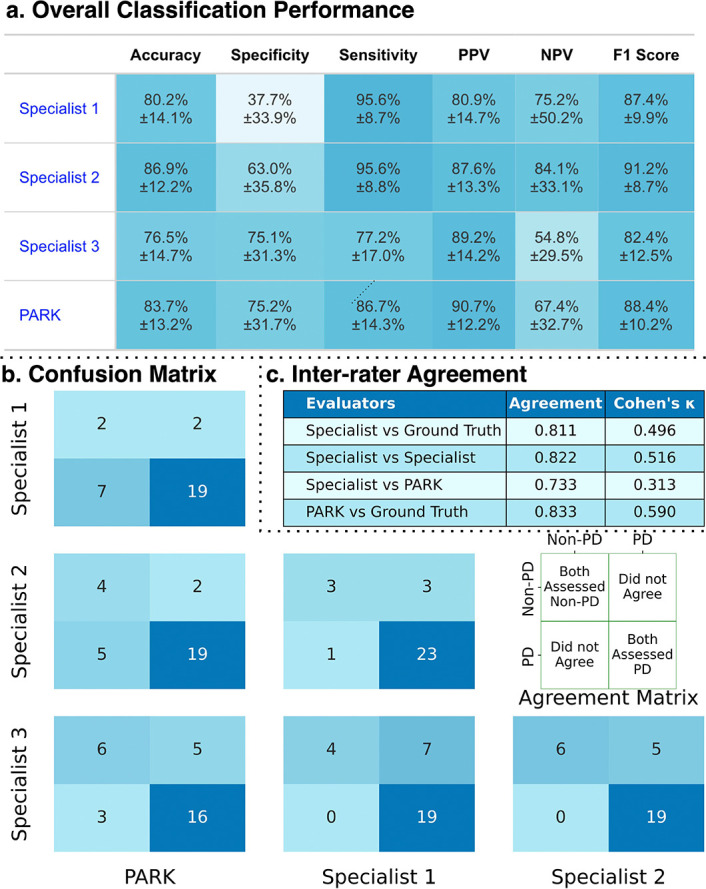
Comparison of PARK with specialist evaluations. (a) Classification performance of PARK and three PD specialists on 30 individuals from the evaluation cohorts with confirmed diagnoses. PARK achieved balanced sensitivity (86.7 ± 14.3%) and specificity (75.2 ± 31.7%), while specialists showed higher sensitivity but lower specificity. (b) Confusion matrices show prediction patterns among the specialist-specialist and PARK-specialist pairs. (c) Inter-rater agreement indicates highest concordance between PARK and ground truth (*κ* = 0.590), with moderate agreement among specialists.

Against ground truth diagnoses, PARK achieved an accuracy of 83.7 ± 13.2% and sensitivity of 86.7 ± 14.3%. The three clinicians achieved accuracies of 80.2 ± 14.1%, 86.9 ± 12.2%, and 76.5 ± 14.7%, with corresponding sensitivities of 95.6 ± 8.7%, 95.6±8.8%, and 77.2±17.0%. Notably, the two clinicians with the highest sensitivity had lower specificity (37.7 ± 33.9% and 63.0 ± 12.2%), reflecting a trade-off favoring recall. In contrast, PARK exhibited more balanced performance across metrics (Fig. 3a). Pairwise Mann-Whitney U Test of bootstrapped distributions across the three clinicians and the clinician majority vote revealed that PARK’s sensitivity, NPV, and *F*_1_ score were significantly lower than those of at least one clinician (FDR-adjusted *p* < 0.05), with NPV also significantly lower than the majority vote (*p* < 0.01). However, PARK’s accuracy, specificity, and PPV did not differ significantly from any individual clinician or their majority vote (FDR-adjusted *p* ≥ 0.527).

Pairwise confusion matrices (Fig. 3b) revealed stronger consensus among the clinicians and PARK for positive predictions, especially in cases where PARK identified PD. This observation is consistent with the high sensitivity and PPV observed across both PARK and the clinicians. Inter-rater agreement analysis (Fig. 3c) further contextualized this alignment. PARK achieved the highest concordance with ground truth diagnoses, with a Cohen’s *κ* of 0.590 and overall agreement of 83.3%. Clinician-clinician agreement was similar (*κ* = 0.516, 82.2%), but alignment with ground truth (*κ* = 0.496, 81.1%) and with PARK (*κ* = 0.313, 73.3%) was lower.

### User-centric Evaluation of PARK

Participants in the external validation cohorts provided structured feedback on the PARK tool across six dimensions: usability, perceived utility, preference relative to traditional clinical visits, perceived risks, perceived benefits, and willingness to interact with the PARK chatbot (developed for responding to individual’s follow-up question upon receiving screening results). Usability and utility were assessed in both supervised and unsupervised cohorts, while the remaining dimensions were evaluated only in the unsupervised setting. The full list of survey items for each category is provided in Extended Data 1. Higher scores indicate more favorable user responses, such as better usability or perceived utility, except for the risk score (lower is better).

Usability was evaluated through Likert-scale items addressing ease of use and user interaction, including the standard 10-item System Usability Scale (SUS) [29], a widely used instrument for assessing digital health tools [30, 31]. The average SUS scores were 70.8 ± 1.3% for the supervised cohort and 75.5 ± 1.6% for the unsupervised cohort, indicating acceptable usability across both environments. Overall usability ratings were high (mean score 4.03/5.00) and consistent across demographic groups, PD status, and supervision level, suggesting broad accessibility. Similarly, perceived utility—whether PARK’s assessments and resources were understandable, informative, and comprehensive—received favorable feedback (mean score 3.96*/*5.00) with consistent ratings across sex and supervision subgroups.

Participants in the unsupervised cohort also rated the perceived risks and benefits of using PARK, as well as their preferences for the tool and the integrated chatbot. On average, users rated the tool as low-risk (0.28/1.00 risk score) and moderately beneficial (3.27/5.00). Participants expressed a general preference for using PARK (average score 3.6/5.00) over in-person clinical appointments when considering specific factors such as accessibility (4.14/5.00), convenience (4.21/5.00), comfort (2.91/5.00), privacy (3.53/5.00), and financial burden (3.20/5.00). However, preferences regarding the LLM-powered chatbot were mixed (average score 2.05/3.00).

User ratings did not significantly differ by sex across any of the six evaluated dimensions (*p* = 0.605,0.662,0.662,0.113,0.955,0.662, respectively, using Mann-Whitney U test), though perceived risk was notably elevated among male participants (*p* = 0.113). Non-white participants expressed more favorable views of PARK across most categories, with statistically significant differences observed in usability, perceived utility, preference for PARK over clinical visits, perceived benefits, and willingness to use the chatbot (*p* = 0.042, 0.003, 0.002, 0.016, and 0.002, respectively). Similar trends were observed among participants without PD, who rated PARK more favorably in terms of usability, perceived utility, preference for PARK over clinical visits, perceived benefits, and willingness to use the chatbot (*p* ≤ 0.002 for all cases). However, participants with PD perceived the risks to be higher than participants without PD (*p* = 0.023).

Finally, participants in the unsupervised Validation Study-2 rated PARK as more usable than those in the supervised Validation Study-1 (*p* = 0.009), while perceived utility did not differ significantly by study supervision status (*p* = 0.582). Note that all the above-reported *p* values are FDR-adjusted using the Benjamini-Hochberg (BH) procedure [32].

### Investigation of Model Errors

**Fig. 5:**
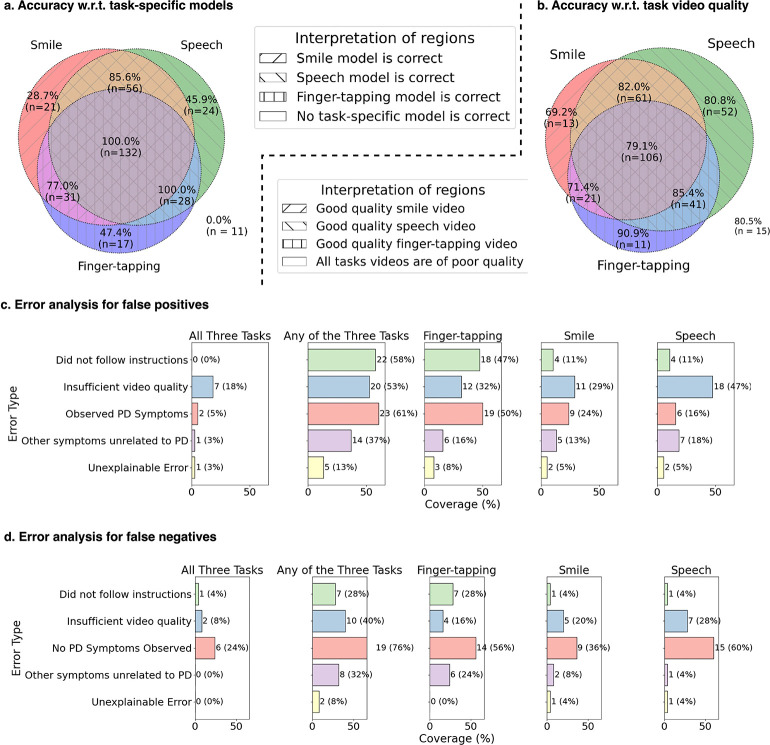
Accuracy decomposition and error analysis of the PARK across tasks and video conditions. (a) Venn diagram annotated with PARK’s prediction accuracy (%) and number of corresponding sessions (n) relative to the accuracy of task-specific models. Each region enclosed by the circles represents cases where one or more task-specific models (smile, speech, or finger-tapping) correctly predicted the PD status. Notably, PARK achieved 100% accuracy when all three task-specific models were accurate, and an accuracy ranging 77.0 – 100.0% even when one of the models were wrong. (b) Corresponding analysis based on task video quality, showing that PARK maintained consistent accuracy across varying levels of video quality. (c) Error analysis for false positives suggests that failure to follow instructions, insufficient video quality, and observed PD symptoms—particularly in the finger-tapping task—despite a negative ground truth, may contribute to erroneous positive predictions. (d) False negatives were most commonly associated with the absence of visible PD symptoms, particularly in the speech and finger-tapping tasks, which together accounted for 56–60% of such errors.

PARK demonstrated consistent ability to integrate signals from the task-specific models (Fig. 5a). As expected, PARK achieved 100 ± 0.0% accuracy when all three task-specific models correctly identified PD status (*n* = 132). However, the tool also maintained high performance when one task-specific model was incorrect. For instance, PARK maintained 100±0.0% accuracy when both the finger-tapping and speech models were correct (*n* = 28), and 85.6±9.0% accuracy when the smile and speech models were correct (*n* = 56). Speech and finger-tapping models were particularly influential–PARK achieved 45.9 ± 20.4% accuracy (*n* = 24) when only the speech model was correct (and other two models were wrong), and 47.4±24.3% accuracy (*n* = 17) when only the finger-tapping model was correct. In contrast, the smile model contributed less to PARK’s final decision.

PARK also performed reliably despite variability in task video quality (Fig. 5b). Note that videos with automatically detected quality issues were excluded from analysis (see Methods: Data Pre-processing). A team of two doctoral and one undergraduate student with 2–5 years of experience in PD video analysis labeled each of the 320 evaluation sessions as having good or poor quality for each task video (see Methods for criteria). PARK maintained 80.5±19.5% accuracy even when none of the task videos were labeled as good quality (*n* = 15), which was comparable to its accuracy when all three task videos were good quality (79.1 ± 7.7%, *n* = 106).

To understand model errors, two doctoral students with 3–5 years of PD video analysis experience manually reviewed all sessions where PARK produced false positives or false negatives. They labeled each task video with possible contributing factors: failure to follow instructions, poor video quality, observable Parkinsonian symptoms in individuals not diagnosed with PD, other non-PD symptoms that may have confused the model, or lack of visible PD symptoms in diagnosed individuals. If none of these factors applied, the video was marked as an unexplained error. All symptom-related annotations were independently re-rated by a neurologist specializing in PD, and their ratings were considered final (and replaced the ratings of the doctoral students).

Among the 38 false positive sessions, a common factor was poor video quality–20 sessions (53%) had at least one low-quality task video, and 7 (18%) had quality issues in all three tasks. Annotators observed Parkinsonian-like symptoms in 23 sessions (61%), with 2 (5%) showing such symptoms across all three tasks. Noncompliance with task instructions was also prevalent, noted in 22 sessions (58%). Only one session (3%) was marked as an unexplained false positive (with no apparent contribution factor for model error).

For the 25 false negative cases, the most frequent issue was the absence of observable PD symptoms–this was noted in 76% of sessions (across one or more tasks) and in 24% of sessions across all tasks. Poor video quality was also associated, with 2 sessions (8%) having inadequate quality across all tasks and 10 sessions (40%) with issues in at least one task.

## Discussion

In this study, we present PARK, an AI-powered screening tool for Parkinson’s disease (PD) that leverages webcam-recorded videos of facial, motor, and speech tasks. PARK integrates uncertainty-aware task-specific classifiers through a lightweight fusion model to deliver accurate predictions even in unsupervised environments. Across three independent cohorts, including a demographically balanced test set and two real-world deployment settings, PARK demonstrated strong and consistent predictive performance. Notably, PARK achieved 80.2–80.6% accuracy, 75.8–81.4% PPV, 78.3–85.3% NPV, 79.4–83.8% *F*_1_-score, and 85.0–87.0% AUROC, while maintaining robustness across demographic subgroups and video quality conditions. In parallel, user-centric evaluations across supervised and unsupervised environments revealed high usability (SUS > 70), broad accessibility, and favorable perceptions across six dimensions, including perceived utility, risks vs. benefits, and preferences over traditional clinical care. These results position PARK as a scalable, user-friendly screening solution with the potential to extend access to neurological assessment in resource-limited or underserved settings. There is a nationwide shortage of clinical neurologists; the shortage is even more acute when it comes to neurologists skilled in the care of patients with movement disorders. One can imagine a patient with potential signs of PD, such as involuntary tremors, undergoing analysis with PARK, with triage decisions based on these results. For such individuals, PARK can serve as a first-line, accessible tool to help them recognize concerning symptoms and seek clinical evaluation sooner. Healthcare systems can reduce long-term economic costs by prioritizing screened individuals and delivering early clinical interventions that enable timely treatment.

From a diagnostic standpoint, PARK achieved high sensitivity (83.3–86.5%) and maintained moderate specificity (71.2–78.4%) across diverse cohorts, suggesting utility in identifying individuals with PD without significantly increasing false alarms. Nonetheless, false positives may lead to unnecessary anxiety, while false negatives may delay care–highlighting the importance of responsible result communication. To mitigate these risks, PARK incorporates uncertainty-aware prediction withholding and communicates results as probabilistic risk scores (not definitive diagnoses), and encourages users to consult medical professionals. These safeguards, combined with strong usability ratings and favorable perceptions reported by end users, support PARK’s safe deployment as a screening—not diagnostic—tool. Its ability to operate reliably across demographic subgroups and video quality conditions further underscores its readiness for equitable, real-world use.

While PARK achieved accuracy, PPV, and *F*_1_ score comparable to three movement disorder specialists, it exhibited higher specificity (75.2 ± 31.7%) and the strongest agreement with ground truth diagnoses (Cohen’s *κ* = 0.590). Clinicians demonstrated higher sensitivity (77.2–95.6%) but lower specificity (37.7–75.1%), likely reflecting a recall-oriented assessment to avoid missed cases. Most discrepancies arose when clinicians predicted PD and PARK did not (Fig. 3b), highlighting this difference in diagnostic behavior. It is important to note that clinicians reviewed only the three recorded videos of facial expression, finger tapping, and speech and did not conduct full clinical assessments due to logistical constraints. Their performance would have been stronger in a traditional in-person clinician-patient encounter. Nevertheless, these findings suggest that PARK can approximate clinician-level performance in focused video-based assessments while offering broader accessibility. Importantly, its decision threshold can be adapted to suit the deployment context—emphasizing sensitivity in low-access regions or prioritizing precision in overburdened systems—making it responsive to both user preferences and clinical demands.

User feedback across two external evaluation studies demonstrated broad acceptability of the PARK tool, with high usability scores (mean SUS: 70.8 ± 1.3% when supervised, and 75.5±1.6% when unsupervised) and favorable ratings across six dimensions of experience. Notably, Non-white participants consistently rated PARK more positively than white participants in usability (FDR-adjusted *p* = 0.042), perceived utility (*p* = 0.003), benefits (*p* = 0.016), preference over clinical visits (*p* = 0.002), and willingness to engage with the chatbot (*p* = 0.002). This disparity may reflect more limited access to traditional neurological care among Non-white individuals, who may perceive PARK as a more viable or empowering alternative. In contrast, participants with PD—most of whom had received a diagnosis and were already connected to care—tended to view the tool as slightly less useful. These findings underscore the importance of considering lived experience and care access when designing and deploying AI screening tools. PARK’s favorable reception among underserved groups suggests it may help reduce disparities in access to neurological assessments, particularly when thoughtfully integrated into existing care pathways.

Open-ended user feedback ranged from enthusiastic endorsement of PARK’s simplicity and clarity to concrete, user-driven suggestions for next-generation refinements. The chatbot comments showed that while some users found it “helpful” and “fast”, others noted repetitive responses and requested richer content links, voice interaction, and smoother hand-offs to a clinician or FAQ when anxiety-provoking topics arose. Across all surveys, participants emphasized embedding clear disclaimers and actionable next-step guidance—such as recommending a neurologist for follow-up consultation or explaining probability scores—to prevent undue alarm. Taken together, these insights underscore PARK’s strong foundation in user-centered design and point to straightforward enhancements to further improve accessibility, trust, and real-world utility.

PARK’s primary users are likely to be older adults, who represent the population most affected by Parkinson’s disease [33]. Given that many may use the tool independently at home, variability in recording conditions—including background noise, lighting, and camera positioning—is inevitable. Despite these challenges, PARK maintained stable performance across a range of video quality levels (Fig. 5b), underscoring its robustness to the kinds of imperfections commonly encountered in real-world environments. This resilience is critical for ensuring reliable assessments in unsupervised, at-home settings.

Manual review of false positive cases revealed that failure to follow task instructions was a common contributing factor, occurring in 58% of such instances–most frequently during the finger-tapping task (47%). In several cases, annotators also observed Parkinsonian symptoms in individuals who did not report a PD diagnosis. Given that the data were collected via a publicly accessible web platform [34], it is possible that some users tested the system by mimicking PD-like symptoms, inadvertently confusing the model. Alternatively, some participants may have had undiagnosed PD, leading to inaccurate self-reporting. While the inclusion of individuals with self-reported diagnoses introduces some uncertainty, it was necessary to ensure broader demographic diversity in the dataset and self-reported PD status has been found to be accurate in remote cohorts [35, 36]. However, we acknowledge the limitations inherent to self-reported clinical status.

Among the false negative cases (*n* = 25), many individuals did not demonstrate PD symptoms observable from the videos. Specifically, in 24% of these cases, no PD symptoms were visible across any of the task videos. Although we carefully selected multiple tasks covering speech, facial expression, and motor assessments, the incorporation of symptom report, additional modalities (e.g., laboratory biomarkers like *α*-synuclein Seed Amplification Assays, or MRI brain scans), or the inclusion of more tasks may help reduce the false negative rate. However, requiring these additional modalities would substantially reduce the accessibility of the PARK tool, as such imaging can be costly and unavailable in underserved communities. Nevertheless, if logistics permit, UFNet can be further trained with the added modalities.

Our study had several limitations. First, data were collected across eight independent studies, each governed by distinct IRB protocols and timelines, resulting in procedural inconsistencies. Notably, different diagnostic criteria were used to confirm PD, some studies (Parktest@InMotion and ROUTE-PD) did not collect demographic information such as ethnicity, and ROOSTER-PD assessed PD severity remotely. While both Validation Study-1 and Validation Study-2 evaluated user experience, Validation Study-1 used only a subset of survey questions. Second, UFNet required all three task videos and could not handle missing data. Although individuals with partial data (*n* = 950, 50.9%) were included in training task-specific models, they could not contribute to advancing the fusion model. Future work may extend UFNet to support incomplete inputs. While this study assembled the largest multi-task video dataset for PD screening to date (1,865 unique participants), a substantial portion of individuals (*n* = 1102,59.1%) self-reported their diagnosis (among 670 individuals with PD, 290 (43.3%) were self-reported). Although participants were recruited globally from 33 countries, the majority (*n* = 1751, 93.9%) were based in the United States. Finally, despite the diversity of our dataset, many subgroups assessed for analyses had small sample size, limiting the power of the corresponding statistical tests. Therefore, some findings may reflect insufficient statistical power rather than a true effect.

In summary, this study introduces a clinically grounded and user-aligned framework for AI-driven remote screening of Parkinson’s disease. By validating PARK across diverse cohorts, deployment conditions, and usability contexts, we demonstrate that scalable, multimodal tools can achieve clinician-comparable performance while remaining accessible to underserved populations. In addition to technical performance, real-world adoption of digital health tools depends on usability, equity, and safety. PARK addresses these dimensions through robust design choices, including task-specific uncertainty modeling, and user-informed evaluation. As healthcare systems increasingly look toward remote and preventive care, tools like PARK offer a viable path forward, lowering barriers to initial neurological assessment and informing future development of inclusive digital health technologies.

## Methods

### Description of Data Sources

Data used in this work were compiled from eight independent studies described below. Each study received approval from the institutional review board (IRB) of the corresponding institution(s).

1. **Parktest (2017–Ongoing)**: Parktest is a non-clinical study aimed at collecting multimodal video and audio recordings from individuals with and without Parkinson’s disease (PD) via a publicly accessible web-based platform[34] (https://parktest.net). Participants were recruited globally through multiple channels, including the University of Rochester Brain Health Registry, clinician referrals, and social media outreach, ensuring broad geographic and demographic representation. Enrollees completed multiple standardized tasks, including the three analyzed in this study, designed to mimic components of the MDS-UPDRS [23]. All recordings were conducted at home without supervision, and instructional videos were provided before each task. Given the non-clinical nature of the study, clinical status (PD or Non-PD) was entirely self-reported and not clinically confirmed.
2. **SuperPD (2020–2022)**: SuperPD was a longitudinal clinical study investigating PD progression over a two-year period. Participants with and without PD were enrolled from the greater Rochester, New York area through the University of Rochester Medical Center (URMC). Participants completed standardized tasks at baseline, 6, 12, and 24 months using the same web-based platform. Therefore, each individual could contribute up to four data sessions across the study period. Data were collected under supervision, and all PD diagnoses were confirmed using the UK Parkinson’s Disease Society Brain Bank Diagnostic Criteria [37].
3. **ROOSTER-PD (2021–Ongoing)**: ROOSTER-PD is a longitudinal sub-study embedded within a six-year remote observational study of individuals carrying the LRRK2 G2019S variant, which is the most common autosomal dominant cause of PD [38]. Participants in the parent study, VALOR-PD [39], were recruited nationwide through 23andMe (https://23andme.com). Participants enrolled in the ROOSTER-PD sub-study complete annual remote assessments using the web platform, with study staff providing supervision as needed during video data collection. Participants included non-manifest carriers without a clinical PD diagnosis and manifest carriers with a clinical PD diagnosis.
4. **Cluster-PD (2021–2023)**: Cluster-PD was a cross-sectional clinical study evaluating environmental exposure as a potential PD risk factor. The study enrolled attorneys from Monroe County (New York). A subset of the attorneys worked adjacent to a site with known trichloroethylene (TCE) and perchloroethylene (PCE) contamination, which are associated with an increased risk of PD [40, 41]. Participants completed the standard PARK tasks under clinical supervision, and PD diagnoses were verified using the Gelb Diagnostic Criteria [42].
5. **Parktest@InMotion (2022–2023)**: For this study, we specifically reached out to the clients of InMotion (a PD wellness center in Ohio, United States) with a clinically confirmed PD diagnosis. We collected their informed consent and asked them to complete the standard PARK tasks. A control group of individuals without PD (clients’ caregivers) was also recruited. Participants completed the tasks onsite and were supervised by the staff at the InMotion facility as needed.
6. **ROUTE-PD (2023–2024)**: ROUTE-PD was a one-year longitudinal study conducted at InMotion, aimed at tracking symptom progression through monthly assessments. Participants with and without PD completed the standard PARK tasks, primarily without supervision. On-demand assistance from staff was available. All PD diagnoses were confirmed clinically at enrollment and re-evaluated at study conclusion using the MDS Clinical Diagnostic Criteria for Parkinson’s Disease [43].
7. **Validation Study-1 (2022–2023)**: Validation Study-1 was conducted to evaluate the usability and real-world performance of the PARK tool in a supervised community setting. InMotion clients (with PD) and their caregivers (without any diagnosed movement disorder) who did not participate in any prior studies relevant to this work were invited to enroll, ensuring our model did not see any data from these participants during training and development. In addition to completing the standardized tasks, participants completed usability surveys (qualitative and quantitative) and provided qualitative feedback on potential risks and benefits of the PARK tool. All participants were supervised by the study staff, and participants with PD had their diagnoses confirmed by a movement disorders neurologist.
8. **Validation Study-2 (2024–2025)**: Validation Study-2 was a replication of Validation Study-1 conducted in an unsupervised, home-based setting. Participants with and without PD were recruited nationwide (within the United States) and completed the standardized tasks and follow-up surveys using the web platform. In addition to the survey questions used in Validation Study-2, we included Likert scale [44] questions regarding potential risks and benefits of the PARK tool, to assess these aspects quantitatively. Inclusion criteria included the ability to provide informed consent and either a PD diagnosis (confirmed by a movement disorders neurologist) or self-reported absence of any movement disorder. Prior participation in PARK studies was an exclusion criterion to ensure the model was tested on unseen individuals. All sessions were completed independently, without supervision.

### Data Pre-processing

Many videos were recorded in unsupervised home environments, resulting in varying levels of quality and compliance. While not all quality issues could be automatically detected, we applied several exclusion criteria to ensure data integrity. Specifically, we automatically discarded videos with the following quality issues:

- videos with abnormal durations (defined as more than three standard deviations below or above the average duration of the respective task videos),
- videos in which the relevant body part was not visible (e.g., obscured hands during the finger-tapping task),
- videos unreadable by the FFmpeg library [45], and
- videos from which task-relevant computational features could not be extracted.

Participants and their data were removed if they did not contribute at least one task-video (not necessarily all three tasks) that satisfied these quality criteria. All remaining videos were standardized to 15 frames per second (fps), resized to 640×480 resolution, and encoded in MP4 format (H.264 codec).

### Dataset Splits

We used data from the first six studies (Parktest, SuperPD, ROOSTER-PD, ClusterPD, Parktest@InMotion, and ROUTE-PD) for model training, selection, and evaluation. From this pool, we randomly sampled 120 participants for model selection, and another 200 for testing, ensuring class balance (PD vs. non-PD) and broad demographic coverage. The remaining participants were assigned to the training set.

To ensure balanced demographic representation, we used a genetic algorithm [46] to maximize diversity within each sampled cohort (except for the training set). We initialized the algorithm with a random set of 50% PD and 50% non-PD participants. We enforced a random, artificial mutation at each iteration–replace a random participant within the set with another random participant (with same PD/non-PD classification) outside the set. The mutation was accepted if it increased demographic diversity of the participants. Specifically, for each set, we defined an expected distribution of different sex (50% male, 50% female), ethnicity (45% white, 45% Non-white, and 10% unknown), and age (45% below 50 years, 45% above 50 years, and 10% unknown) subgroups. Then we proposed a diversity loss ${ℒ}_{\mathrm{diversity}}$ that penalized when the observed numbers in each subgroup differed from the expected numbers.

(1) $${ℒ}_{\mathrm{diversity}}=\sum_{a\in C}\sum_{i\in a}\frac{{\left(E_{i}\;-\;O_{i}\right)}^{2}}{E_{i}}$$

Here, C are different demographic attributes (i.e., sex, ethnicity, and age), i are the subgroups for an attribute a (e.g., male, female), and $E_{i}$, and $O_{i}$ are the expected, and observed number of participants, respectively, for the target subgroup. Smaller differences are less penalized, while the larger differences are heavily penalized due to the quadratic nature of ${ℒ}_{\mathrm{diversity}}$. Reduced loss corresponds to increased demographic diversity. Eventually, the genetic algorithm trying to minimize this loss converges and reaches a local optima. To approximate the global optimum, the algorithm was run 500 times and the solution with the minimum diversity loss was chosen. First, 200 participants (100 with PD vs. 100 without; 49% male vs. 51% female; 45% white vs. 24% Non-white and 31% unknown; 29% below 50 years vs. 59% above 50 years and 12% unknown) were chosen for model testing (termed as Balanced Test Data throughout the article) out of the entire population of six studies. Then, the chosen 200 participants were excluded and the entire process was re-run to select the 120 participants for model selection.

Participants from two separate validation studies (Validation Study-1 and Validation Study-2) were reserved for real-world evaluation of the final (locked) model. These are referred to as External Evaluation (Supervised) and External Evaluation (Unsupervised), respectively, throughout this paper.

### Task Selection

Parkinsonian symptoms can be multi-faceted and are often expressed across multiple modalities–facial expressions, motor functions, and speech. To analyze multiple modalities simultaneously, we collected audio and video recordings of three standardized tasks: finger tapping (motor function), smile mimicry (facial expressions), and pangram^1^ utterance (speech). The finger-tapping task requires participants to tap their thumb with the index finger ten times, performing the motion as fast and big as possible. In the smile mimicry task, they are instructed to smile naturally in front of the camera, then gradually return to a neutral facial expression, repeating this sequence three times. In the pangram utterance task, participants are asked to read aloud a sentence that contains all the letters of the English alphabet. For this study, we used the widely-used [14, 15] pangram: “The quick brown fox jumps over the lazy dog.”

The selection of these tasks was guided by considerations of clinical relevance, feasibility for at-home deployment, and participant safety. Although gait analysis is a well-established for assessing PD severity and progression [11, 47], its remote implementation poses significant challenges. Accurately capturing gait requires full-body visibility, adequate walking space, and controlled environments, all of which are difficult to guarantee in unsupervised home settings. Moreover, the physical exertion involved may increase the risk of falls among individuals with advanced motor symptoms. Similarly, sustained phonation–where participants are asked to hold a vowel sound for as long as possible–has shown promise in PD detection [48, 49]. However, its utility is often hindered by variability in microphone sensitivity, and ambient noise, complicating downstream acoustic analysis. In contrast, the selected tasks can be performed while seated, require minimal equipment, and are robust to environmental variations, making them well-suited for scalable, at-home screening [24].

### Extracting Computational Features

Deep learning models have shown remarkable success in learning representations from raw, non-tabular inputs such as video and audio. However, their performance typically depends on access to large-scale, annotated datasets; for example, state-of-the-art cancer detection systems are often trained on hundreds of thousands of samples [50]. Given the limited size of our dataset of patient-recorded videos, we adopted a feature engineering approach informed by prior literature and domain expertise. Rather than relying solely on data-intensive deep learning models, we extracted clinically meaningful, task-specific features tailored to each standardized neurological task—finger tapping, facial expression, and speech. These features have been validated in previous studies and are robust to the recording variability observed across both home and clinical settings. While we refer the reader to previous work for detailed definitions and validation of these features, we briefly summarize them below.

#### Finger-tapping Task:

Inspired by Islam et al. [18], we utilized MediaPipe [25] to extract 65 features per hand to objectively analyze bradykinesia [51] and rigidity through features such as tapping speed, amplitude, and interruptions. This extraction process resulted in a total of 130 features for the finger-tapping task to model motor impairments.

#### Smile Task:

Adnan et al. [52] identified 42 facial features for analyzing smile mimicry videos. These features, extracted using OpenFace [26] and MediaPipe, captured digital markers for PD closely aligned with the MDS-UPDRS [23] guidelines for in-person evaluation, such as intensity of facial muscle movements, smile spontaneity, eye blinking, lip separation, etc. Machine learning models trained solely on this feature set demonstrated promising performance, indicating the effectiveness of these features in detecting PD-related changes in facial expressions (such as hypomimia [53]).

#### Speech (Pangram Utterance) Task:

For the speech task, we extracted 1024-dimensional embeddings using WavLM [27], a self-supervised speech representation model pre-trained on a large-scale, diverse audio corpus. These embeddings capture fine-grained acoustic features—including prosody, articulation, and vocal quality—that are frequently impaired in individuals with PD. In our prior work [15], we demonstrated that WavLM-based embeddings significantly outperformed conventional handcrafted features such as pitch, jitter, and shimmer, highlighting their utility for PD screening in real-world, unconstrained environments.

### Model Training

We followed the training protocol established in our prior work [24], which involves a two-stage pipeline: task-specific modeling followed by multimodal fusion using the Uncertainty-calibrated Fusion Network (UFNet). Task-specific models were trained using all available data from training set participants. For individuals who contributed multiple sessions across time—particularly in longitudinal studies like ROOSTER-PD—each session was labeled based on the most recent PD diagnosis status preceding the session date. UFNet was trained exclusively on sessions where all three task videos (motor, facial, and speech) were present and met quality criteria.

In the first stage, each of the three tasks–finger-tapping, smile, and pangram utterance–is independently modeled using shallow neural networks tailored to the domain-specific features. All task-specific models were trained with binary cross-entropy loss, employed ReLU activations (with sigmoid on the output), and used either SGD or AdamW optimizers. Importantly, Monte Carlo (MC) dropout was applied during both training and inference to quantify uncertainty by averaging predictions over multiple stochastic forward passes.

In the second stage, the UFNet model integrates the outputs from the three task-specific networks in an uncertainty-aware fashion. Each task’s features are first projected to a shared dimensional space using linear transformations followed by ReLU activation, MC dropout, and layer normalization. These projected vectors are then combined using a calibrated self-attention mechanism, where attention weights are modulated by task-specific uncertainties to down-weight unreliable modalities. The contextualized representations from self-attention, along with the mean prediction probabilities from the task-specific models, are concatenated and passed through another shallow neural network trained with MC dropout. To ensure safe deployment in clinical screening settings, UFNet incorporates a mechanism to withhold predictions for uncertain cases. Specifically, predictions are flagged as uncertain if the 95% confidence interval from MC dropout includes both PD and Non-PD classes, preventing potentially misleading outputs.

This two-stage architecture enables the model to robustly integrate heterogeneous signals from motor, facial expression, and speech modalities while accounting for task-specific reliability. Also, UFNet only has 226,000 trainable parameters, making it suitable for training with smaller datasets and deployment in lightweight environments such as personal computers.

### Model Selection and Performance Reporting

Both the task-specific models and the UFNet employ shallow neural network architectures and require tuning of several hyperparameters, including learning rate, batch size, dropout probability, and optimizer settings. Given the class imbalance in the training set (more Non-PD than PD samples), we also explored the utility of synthetic minority oversampling techniques such as SMOTE [54]. Task-specific features were further processed through multiple configurations of feature scaling (e.g., standardization, min-max normalization) and feature selection (e.g., correlation-based filtering). We employed Weights & Biases (https://wandb.ai) to conduct an efficient hyperparameter search (see Extended Data 2 for the complete search space), using Bayesian optimization to maximize the Area Under the Receiver Operating Characteristic (AUROC) on the model selection dataset.

Due to the two-stage architecture, we first performed hyperparameter tuning independently for each task-specific model, selected the best-performing configuration on the model selection set, and then locked those models. The outputs from these locked task-specific models were then used as inputs to UFNet, for which a separate round of hyperparameter tuning was conducted. The final UFNet model–selected based on highest AUROC on the model selection set–was evaluated on the balanced test data and the two external validation sets (supervised and unsupervised) independently.

Model performance was assessed using metrics critical for clinical evaluation: AUROC, Area Under the Precision-Recall Curve (AUPRC), Accuracy, *F*_1_-score, Sensitivity, Specificity, Positive Predictive Value (PPV), and Negative Predictive Value (NPV). To assess the reliability of model predicted probabilities, we also evaluated calibration using Expected Calibration Error (ECE) [55] and Brier Score [28], ensuring that model predictions align with the true likelihood of Parkinsonian disorder.

### Specialist Assessments

To benchmark the PARK tool’s performance against clinical expertise, we conducted a comparative evaluation using a curated set of 30 participants randomly drawn from the validation cohorts (Balanced Test Data, External Evaluation (supervised), and External Evaluation (unsupervised)). All participants had completed the full set of standard PARK tasks (finger-tapping, smile, and speech).

Three experienced movement disorder specialists–each an associate or full professor in the Department of Neurology at the University of Rochester Medical Center with over a decade of clinical experience diagnosing and managing Parkinsonian disorders–independently reviewed these recordings. However, full in-person clinical assessments were not feasible for this cohort due to logistical and ethical considerations. Participants were geographically dispersed and recruited remotely, making it difficult to trace and invite them for follow-up assessments. Moreover, re-contacting participants solely for clinical evaluation was not covered under the original IRB protocols. As a result, the specialists based their evaluations exclusively on the video recordings of the three standardized tasks, without access to clinical history or neurological exams. This constraint mirrors the real-world deployment setting of the PARK tool, which also operates solely on task recordings.

Each specialist independently assessed whether the participant exhibited symptoms consistent with a Parkinsonian disorder. The same recordings were simultaneously processed by the PARK model, which had been locked prior to evaluation. Performance was quantified using standard diagnostic metrics–accuracy, sensitivity, specificity, positive predictive value (PPV), negative predictive value (NPV), and *F*_1_ score. To contextualize PARK’s performance relative to clinical judgment, we also computed inter-rater agreement among the specialists and between each specialist and the PARK tool using Cohen’s kappa.

### Development of PARK Chatbot

To complement the AI-based remote screening provided by the PARK tool, we developed an LLM-driven chatbot (PARK chatbot), designed to support participants immediately following the presentation of their screening results. The chatbot was was built on a GPT-3.5 backend and integrated into the web platform used in both Validation Study-1 and Validation Study-2. The chatbot served as an interactive guide to help users interpret the screening outcomes, access curated educational materials, and identify local resources for follow-up care. Specifically, it was configured to explain screening results using plain language and point users to trusted resources, including Movement Disorder Society (MDS) directory for neurologists and local support groups.

To avoid any potential distress, we added guardrails to the chatbot to ensure it offers consistent and appropriate responses, and does not provide clinical advice (such as medication). To achieve this, the system was constrained through a set of strict behavioral instructions that enforced the following:

- No responses to questions about medications or treatment plans
- No interpretation of symptoms as medical diagnosis
- No provision of clinic-specific or time-sensitive information
- No engagement in off-topic dialogue

In these cases, the chatbot provided links to verified external sources such as the Parkinson’s Foundation. In addition, the chatbot is designed to reiterate that the PARK tool is a screening tool–not a diagnostic platform–and that all health decisions should be made in consultation with a qualified clinician. The guardrails were enforced through a structured prompt.

### User-centric Assessments

To evaluate the usability, perceived benefits and risks, and user preferences associated with the PARK tool when used in home or community environments, we conducted two independent user studies—Validation Study-1 and Validation Study-2. In addition to capturing video recordings of participants performing standard PARK tasks, we administered structured surveys to quantify user feedback across multiple dimensions. The surveys were designed after discussing with multiple stakeholders associated with PD care: three neurologists, individuals with PD and their caregivers (without PD), and a bio-ethics specialist.

Validation Study-1 was conducted in a supervised community setting where participants interacted with the PARK tool while receiving assistance from onsite staffs familiar with the task requirements and technology. In contrast, Validation Study-2 was designed to reflect real-world conditions more closely, where participants completed the protocol independently in their home environments without direct supervision.

Note that, due to protocol variances and differing timeline in these two studies, quantitative responses to some of the sections (marked with a *) were only collected in Validation Study-2, while Validation Study-1 asked open-ended questions to assess these categories. Although responses to the open-ended questions helped us gather insights, they were not included in the quantitative analyses discussed in this paper. The detailed list of the survey questionnaires are documented in Extended Data 1.

- **Usability.** All participants completed a series of Likert-scale survey items assessing usability and system functionality, including a modified System Usability Scale (SUS). Questions addressed ease of task completion, clarity of instructional materials, and the intuitiveness of interacting with PARK chatbot (e.g., “The instructional videos helped me to complete the tasks”, “The tasks were easy to complete”, “It was easy to interact with PARK-Bot”).
- **Utility and Comprehensiveness of Resources:** To evaluate the utility and comprehensiveness of educational and interpretive resources provided by the tool, participants responded to questions concerning the clarity and informativeness of risk descriptions and severity ratings, as well as the adequacy of answers provided by PARK-Bot.
- Preference Compared to Clinical Appointments*: Participants compared their experience using PARK to traditional in-person clinical assessments in terms of accessibility, convenience, comfort, privacy, and financial impact.
- Perceived Risks and Benefits*: Questions in this section captured emotional responses to the PARK results, including feelings of anxiety or empowerment, and concerns regarding the potential harm of automated responses.
- Preference for Using PARK chatbot*: Participants indicated their preferences for interacting with the conversational agent across different contexts (e.g., for ease-of-use, comfort, or safety).

Survey responses provided insight into the real-world usability and perceived value of PARK across different supervision conditions, helping to identify barriers and facilitators to adoption in diverse settings.

### Statistical Analysis

All statistical analyses were conducted using Python (version 3.11.7) with standard scientific libraries such as SciPy, NumPy, and statsmodels. Performance metrics for classification tasks—including accuracy, sensitivity, specificity, PPV, NPV, AUROC, and Brier score—were reported with 95% confidence intervals (CIs), computed via bootstrapped resampling with replacement (*n* = 1,000).

To compare model performance across test cohorts (Balanced Test Data, External Evaluation (Supervised), and External Evaluation (Unsupervised)) and against clinician ratings, we applied the non-parametric Mann–Whitney U test on the bootstrapped performance distributions. This test was selected because it does not assume normality and is appropriate for comparing independent, resampled metric distributions. To evaluate potential demographic biases in model performance by sex and race, we used Z-tests for proportions, applied only when the sample size met the standard assumptions (*np* ≥ 5 and *n*(1–*p*) ≥ 5). For comparisons involving categorical variables such as age groups or Hoehn and Yahr PD stages, we applied chi-square tests of independence. Since the expected cell frequency assumption (≥ 5) was violated in the PD stage-based analysis, we used a Monte Carlo simulation with 10,000 replications to estimate p-values for the chi-square statistic, ensuring valid inference in the presence of sparse data. Since we conducted multiple statistical test on the same model predictions, which inflates the risk of Type I error, we applied a multivariable logistic regression analysis followed by Benjamini–Hochberg False Discovery Rate (FDR) correction to adjust the resulting p-values.

For assessing model calibration, Brier scores were computed and calibration curves were generated by plotting the predicted PD probabilities against the observed outcome frequencies across bins. For inter-rater agreement across PARK predictions, clinician annotations, and the ground truth labels, we calculated Cohen’s *κ* coefficient. In the usability study involving the Likert-scale responses (treated as ordinal or non-normally distributed continuous data), comparison between demographic subgroups were conducted using the Mann-Whitney U test with FDR correction applied using the Benjamini–Hochberg procedure. All statistical significance tests were evaluated at a threshold of *p* < 0.05 after FDR correction, unless otherwise specified.

### Video Quality Analysis

To ensure data integrity and the reliability of downstream analyses, we conducted a comprehensive quality assessment of all video recordings, including audio, smile, and finger-tapping tasks. Three annotators–two doctoral students and one undergraduate student with 2–5 years of experience in PD video analysis performed the evaluations. All annotators had 2+ years of experience developing and interacting with the PARK framework, had knowledge regarding clinical PD assessments and MDS-UPDRS criteria, and were well-acquainted with the dataset, ensuring credibility and consistency.

In the initial phase, annotators independently assessed 50 random recordings of each task, and rated video quality in a three-points scale: 0 (poor quality), 1 (ambiguous), and 2 (high quality). They then met to resolve discrepancies and establish a standardized guideline to maintain labeling consistency. This process facilitated a set of clearly defined criteria for the annotations (see Extended Data 3). In summary, for audio recordings, we evaluated background and microphone noise, unnatural pauses, and verbal compliance, without incorporating visual features. In contrast, smile and finger-tapping videos were assessed based on visibility, background contrast, participant positioning, and adherence to task instructions. Final quality annotations of the videos used for evaluating PARK were based on the collaboratively developed guidelines. The “ambiguous” category was applied sparingly and only in cases with conflicting quality indicators.

## Extended Data

**Extended Data 1:** Survey questions used to evaluate usability, risk, benefit, and user preferences regarding the PARK tool.

**Survey Questions Administered to Participants (Response formats indicated per section)**
**Usability**
1: Strongly Disagree / 2: Disagree / 3: Neutral / 4: Agree / 5: Strongly Agree
1. The instructional videos helped me to complete the tasks.
2. The tasks were easy to complete.
3. It was easy to interact with PARK-Bot.
4a. I think that I would like to use the PARK tool frequently.
4b. I found the PARK tool unnecessarily complex.
4c. I thought the PARK tool was easy to use.
4d. I think that I would need the support of a technical person to be able to use the PARK tool.
4e. I found the various functions in the PARK tool were well integrated.
4f. I thought there was too much inconsistency in the PARK tool.
4g. I would imagine that most people would learn to use the PARK tool very quickly.
4h. I found the PARK tool very cumbersome to use.
4i. I felt very confident using the PARK tool.
4j. I needed to learn a lot of things before I could get going with the PARK tool.
**Utility and Comprehensiveness of Resources**
1: Strongly Disagree / 2: Disagree / 3: Neutral / 4: Agree / 5: Strongly Agree
5. The description of the risk of demonstrating features of Parkinson’s disease was easy to understand.
6. The description of the risk of demonstrating features of Parkinson’s disease was informative.
7. The severity ratings provided for the speech, facial expression, and motor tasks were easy to understand.
8. The severity ratings provided for the speech, facial expression, and motor tasks were informative.
9. PARK Chatbot adequately answered my questions.
10. The resources provided were informative.
11. The resources provided were comprehensive.
**Preference Compared to Clinical Appointments**
1: Less Preferred / 2: Somewhat Less Preferred / 3: About the Same / 4: Somewhat More Preferred / 5: More Preferred
12. In terms of accessibility, how does using the PARK tool compare to having an appointment at a medical facility?
13. In terms of convenience, how does using the PARK tool compare to having an appointment at a medical facility?
14. How would you rate your level of comfort when using the online assessment tool compared to being assessed in a medical facility?
15. How would you rate your level of privacy when using the online assessment tool compared to being assessed in a medical facility?
16. I would prefer the overall financial impact of using an online tool compared to visiting a medical facility.
**Perceived Risks from Using the PARK Tool**
0: No / 1: Yes
17. Viewing the results from PARK caused me to feel anxious.
18. Do you think the PARK tool responses could be harmful?
19. Do you think the PARK Chatbot responses could be harmful?
**Perceived Benefits from PARK Tool**
1: Strongly Disagree / 2: Disagree / 3: Neutral / 4: Agree / 5: Strongly Agree
20. Viewing the results from PARK caused me to feel empowered.
21. Viewing the results from PARK caused me to feel in control of my health.
**Preference for using PARK Chatbot**
1: Never / 2: Sometimes / 3: Often
22. In terms of comfort, when would you prefer to speak to PARK Chatbot?
23. In terms of comprehensiveness, when would you prefer to speak to PARK Chatbot?
24. In terms of ease-of-use, when would you prefer to speak to PARK Chatbot?
25. In terms of safety, when would you prefer to speak to PARK Chatbot?
26. In general, when would you prefer to speak to PARK Chatbot?

**Extended Data 2:** Hyper-parameter search space for the UFNet models.

Hyperparameter	Values/range	Distribution

batch size	{256, 512, 1024}	Categorical
learning rate	[5*e*^−5^,1.0]	Uniform
use minority oversampling?	{“yes”, “no”}	Categorical
oversampling method	{“SMOTE”, “SVMSMOTE”, “ADASYN”, “BoarderlineSMOTE”, “SMOTEN”, “RandomOversampler”}	Categorical
number of hidden layers	{1}	Categorical
projection dimension	{128, 256, 512}	Categorical
query (query/key/value) dimension	{32, 64, 128, 256}	Categorical
hidden dimension	{4, 8, 16, 32, 64, 128}	Categorical
dropout probability	[0.05, 0.50]	Uniform
*η* (UFNet’s internal parameter)	[0.1, 100]	Uniform
number MC dropout rounds	{30}	Categorical
number of epochs	[1, 300]	Uniform Integer
optimizer	{“SGD”, “AdamW”, “RMSprop”}	Categorical
momentum	[0.1,1.0]	Uniform
use scheduler?	{“yes”, “no”}	Categorical
scheduler	{“step”, “reduce on plateau”}	Categorical
step size	[1, 30]	Uniform Integer
patience	[1, 20]	Uniform Integer
gamma	[0.5, 0.95]	Uniform

**Extended Data 3:** Criteria used to assess video quality. Either a red item or combination of multiple items in the list meant a video is of poor quality. A single black item meant the quality label was ambiguous. Absence of all the items meant good quality video.

**Quality Assessment Criteria**
**Finger-tapping Task**
1. Blurry or dark video, but hand is clear
2. Hand is unclear due to blurriness and poor contrast
3. Multiple persons’ hands are visible but only participant is tapping
4. Multiple persons’ hands are visible and the person(s) in the background is tapping
5. Participant seated far from the camera – chest/waist visible
6. Participant seated too far from the camera – knees/legs visible
7. Wrist partially/fully goes out of frame for a few moments, but 5+ taps with consecutive wrist visibility
8. Wrist goes out of frame for over 2 seconds during the tapping task
9. Taps 3–4 times less/more than instructed
10. Taps 5+ times less/more than instructed
11. **Miscellaneous noncompliance:** video recorded from phone, bad angle (not front-facing the camera), shaky camera, stayed idle entire time, did not attempt the task
**Smile Task**
1. Idle for over five seconds before/after task.
2. Idle for over five seconds during task completion
3. Blurry or dark video, but face clear
4. Face unclear
5. Multiple persons’ faces visible
6. Minor glare from eyeglasses, but eyes remain visible
7. Major glare blocks eye
8. Laughing or smile too fast
9. Some body movement, but face remains in the frame
10. Face goes out of frame due to body movement
11. Participant seated far from the camera – chest/waist visible
12. Participant seated too far from the camera – knees/legs visible
13. **Miscellaneous noncompliance:** video recorded from phone, talking during the task, bad angle (not front-facing the camera), face not in frame, shaky camera, stayed idle entire time, did not smile
**Speech Task**
1. Noise but speech clear
2. Speech hard to hear
3. Unnatural short pauses, trying to remember the phrase
4. Unnatural long pauses and irrelevant words/sentences
5. 3+ repeated words
6. Utterance said multiple times
7. Missed 3–5 consecutive words
8. Missed entire sentence
9. Multiple speakers

## Figures and Tables

**Fig. 4:**
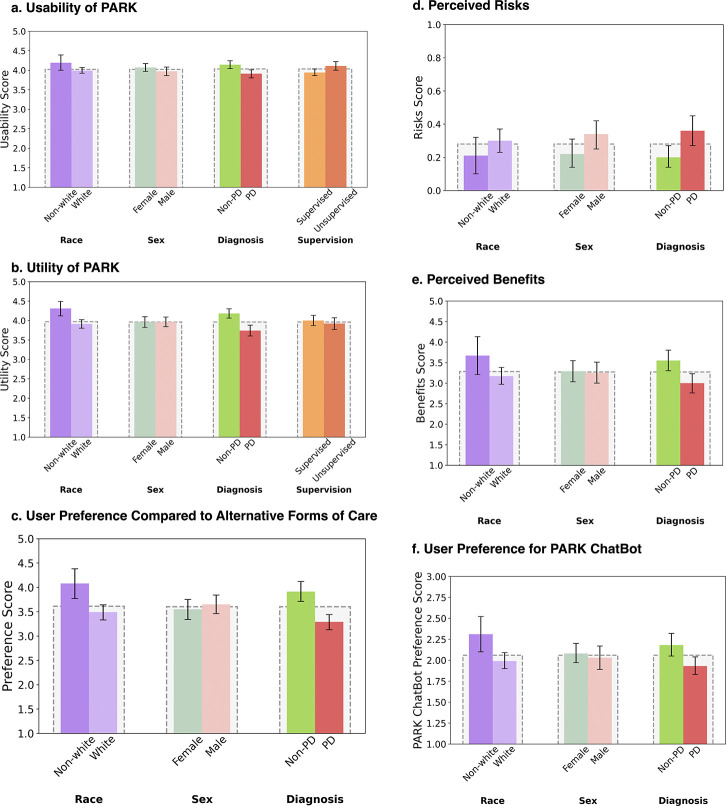
Usability, perceived value, and user preferences for the PARK screening tool across demographic subgroups. Survey responses were aggregated by category and stratified by race, sex, diagnosis status, and supervision setting. Questions on perceived risks were binary (e.g., whether PARK caused anxiety or its responses were potentially harmful), while preferences for the chatbot were rated on a three-point Likert scale (often, sometimes, never). All other items used a five-point Likert scale (strongly disagree, disagree, neutral, agree, strongly agree). Lower risk score is better, while for other categories, higher scores indicate more favorable responses. For each category, Likert responses were averaged to compute the final score. (a) Usability scores were consistently high across demographic subgroups. (b) Perceived utility of PARK was similarly favorable across most subgroups. (c) Preference for PARK over traditional care was higher among Non-white and non-PD participants. (d) Perceived risks were low overall but elevated among individuals with PD. (e) Perceived benefits were rated more favorably by Non-white and non-PD users. (f) Willingness to interact with the PARK chatbot was moderate overall, with higher favorability among Non-white and non-PD groups. Error bars represent 95% confidence intervals.

## Data Availability

The recorded videos were collected using a web-based tool. The tool is publicly accessible at https://parktest.net. Due to the compliance with the Health Insurance Portability and Accountability Act (HIPAA), we cannot share the raw videos. Nevertheless, the extracted features, class labels (PD/Non-PD), and clinician assessments will be made publicly available (via a GitHub repository) upon the acceptance of the manuscript. The features will be provided in a structured format and with instructions to integrate with existing machine-learning workflows easily.

## Footnotes

1 a sentence containing all the letters of the alphabet
